# To study the role of pre‐treatment microRNA (micro ribonucleic acid) expression as a predictor of response to chemoradiation in locally advanced carcinoma cervix

**DOI:** 10.1002/cnr2.1348

**Published:** 2021-03-03

**Authors:** Soumitra Barik, Swarupa Mitra, Moushumi Suryavanshi, Abhinav Dewan, Inderjeet Kaur, Dushyant Kumar, Maninder Mishra, Gayatri Vishwakarma

**Affiliations:** ^1^ Department of Radiation Oncology Rajiv Gandhi Cancer Institute and Research Centre New Delhi India; ^2^ Department of Molecular Biology Rajiv Gandhi Cancer Institute and Research Centre New Delhi India; ^3^ Department of Biostatistics Rajiv Gandhi Cancer Institute and Research Centre New Delhi India

**Keywords:** biological markers, carcinoma cervix, concurrent chemoradiotherapy, microRNAs, prognosis

## Abstract

**Background:**

Concurrent chemoradiotherapy followed by brachytherapy is the standard of care in locally advanced carcinoma cervix. There is no prognostic factor at present to predict the outcome of disease in locally advanced carcinoma cervix.

**Aim:**

Differential expression of microRNAs can be used as biomarkers to predict clinical response in locally advanced carcinoma cervix patients.

**Methods:**

Thirty‐two patients of locally advanced carcinoma cervix with International Federation of Gynecology and Obstetrics Stage IB‐IVA were enrolled from 2017 to 2018. Expression of microRNA‐9 5p, ‐31 3p, ‐100 5p, ‐125a 5p, ‐125b‐5p, and –200a 5p in formalin‐fixed paraffin embedded (FFPE) biopsied tissue were analyzed by real time quantitative reverse transcriptase polymerase chain reaction (RT qPCR). Pretreatment evaluation was done with clinical examination and MRI pelvis. All patients received concurrent chemoradiotherapy followed by brachytherapy. Patients were evaluated for the clinical response after 3 months of treatment, with clinical examination and MRI pelvis scan using RECIST 1.1 criteria. Patients with no residual disease were classified as Complete responders (CR) and with residual or progressive disease were classified as Nonresponders (NR). Results were statistically analyzed using Mann Whiney *U* test to examine significant difference between the expression of microRNA between complete responders (CR) and nonresponders (NR).

**Results:**

microRNA‐100 5p was upregulated in complete responders (CR) which showed a trend towards statistical significance (*p* value = 0.05).

**Conclusion:**

microRNA‐100 5p can serve as a potential molecular biomarker in predicting clinical response to chemoradiation in locally advanced Carcinoma cervix. Its role should be further investigated in a larger study population.

## 1 INTRODUCTION

Cervical cancer is the fourth most common cancer worldwide with an estimated incidence of 570 000 cases in 2018.^1^ With 31 1000 death in 2018, it is the fourth leading cause of cancer death worldwide. It is the most commonly diagnosed cancer in Sub‐Sharan Africa and Southeastern Asia, where most patients present as locally advanced disease. External beam radiotherapy with concurrent chemotherapy followed by brachytherapy is the standard of care for locally advanced carcinoma cervix.^2^ There is a higher risk of disease recurrence in patients presenting with locally advanced carcinoma cervix. Presently, there is no molecular marker in carcinoma cervix to predict the disease outcome.

MicroRNAs are a family of small noncoding RNA (Ribonucleic Acid) which control translation of messenger RNA (mRNA) into protein by posttranscriptional gene silencing.^3^, ^4^, ^5^ Studies have shown that microRNA regulates thousands of human protein‐coding genes.^6^, ^7^ MicroRNAs regulate many important biological processes such as cell division, cell differentiation, apoptosis, and cancer development.^8^, ^9^, ^10^, ^11^

Many groups have investigated the role of microRNA in cervical cancer, including Wang et al, who reported that microRNA‐143 and ‐145 suppress cell growth and microRNA‐146 promotes cell proliferation in cervical cancer.^12^ Le et al identified that microRNA‐29 acts as a tumor suppresser by inhibiting cell cycle progression and inducing apoptosis through YY1 and CDK6 protein.^13^ Wang et al also reported that human papillomavirus (HPV) induces aberrant expression of many cellular microRNAs. They reported microRNA‐16, ‐25, ‐92a, and ‐378 increasingly expressed and microRNA‐22, ‐27a, ‐29a, and ‐100 decreasingly expressed in HPV infected cells and this could be assigned due to oncoprotein E6 and E7 of HPV.^14^

Though there are reported studies about the role of microRNAs in pathogenesis and carcinogenesis of cervical cancer, there are only a few studies regarding microRNAs predicting the outcome of disease in cervical cancer. Hu et al reported two microRNAs, microRNA‐9, and microRNA‐200a, that could predict survival in patients with carcinoma cervix.^15^ Recently, Pedroza‐Torres et al reported, in patients with locally advanced cervical cancer, microRNA‐31‐3p, ‐3676, ‐125a‐5p, ‐100‐5p, ‐125b‐5p, and ‐200a‐5p and ‐342 were expressed differentially in patients who respond to standard therapy of concurrent chemoradiotherapy than who do not respond to it.^16^

In this study, we examined the expression profile of six microRNAs (microRNA‐9 5p, ‐31 3p, ‐100 5p, ‐125a 5p, ‐125b 5p, and ‐200a 5p) in locally advanced carcinoma cervix. Differential expression of these six microRNAs was compared between patients who had a complete clinical response to standard treatment of chemoradiation to patients who did not respond to it.

## 2 METHODS

Sample size was calculated using the formula (*Z*_(1−alpha)_ × *p*(1 − *p*)/*d*)^2^. With reference to previous studies, (*p*) = 50%, margin of error (*d*) = 8% of *p* and *Z*_(1− alpha)_ = 1.96, a sample size of 38 cases were needed. Thirty eight patients with locally advanced carcinoma cervix with FIGO (International Federation of Gynaecology and Obstetrics) Stage IB‐ IVA were enrolled at our institute from 2017 to 2018 after taking informed written consent from every patient. Approval was taken from scientific and ethical review boards of the institute before starting the study.

Patients with newly diagnosed histologically proven carcinoma cervix, with no coexisting or prior malignancy, who had not received any prior treatment for their cancer, Karnofsky Performance scores more than and equal to 70 were included in the study. Patients with early‐stage (FIGO IA) or metastatic disease (FIGO IVB), patients who had received any prior treatment for carcinoma cervix and or receiving any treatment other than standard therapy were excluded from the study.

After histological confirmation of cervical carcinoma, expression of six microRNA (microRNA‐9 5p, ‐31 3p, ‐100 5p, ‐125a 5p, ‐125b 5p, and ‐200a 5p) in formalin‐fixed paraffin embedded (FFPE) tissue was examined by real‐time quantitative reverse transcriptase‐polymerase chain reaction (RT qPCR).

miRNA extraction was done using Qiagen miRNeasy mini kit (cat no.‐ 217004). The miRNeasy Mini Kit enables purification of total RNA, which includes RNA from approximately 18 nucleotides (nt) upwards from all types of animal tissues and cells, including difficult‐to‐lyse tissues. Alternatively, a miRNA‐enriched fraction and a total RNA (>200 nt) fraction can be purified separately. Purification of miRNA was done as per kit protocol.

All six miRNAs expression was checked on Qiagen Rotor gene Q real‐time PCR using Thermo provided assayed. TaqMan microRNA reverse transcription kit (4366596) was used for cDNA conversion. And TaqMan Universal mastermix was used for expression profile (4440042). We used TaqMan microRNA assay (by Thermo Fischer Scientific^R^) for our study. Catalog number of the six microRNAs assay used were as follows, miRNA 9‐5p (478214), miRNA 31‐3p (478012), miRNA 100‐5p (478224), miRNA 125a‐5p (477884), miRNA 125b‐5p (477885), and miRNA 200‐5p (478752).

The expression of microRNAs was calculated by double delta Ct (threshold cycles) method.^17^

*Double delta CT method*:

TE‐ CT value of test miRNA in Cases

TC‐ CT value of test miRNA in control

HE‐ CT value of physiological miRNA in Cases

HC‐ CT value of physiological miRNA in control.

DELTA CT_E_ = TE ‐ HE

DELTA CT_C_ = TC ‐ HC

Double DELTA = DELTA CT_E_‐ DELTA CT_C_

Expression in fold changes = 2̂^‐(Double DELTA)^.

For calculation by the double delta Ct (threshold cycle) method, the expression of six aforementioned microRNAs was examined in seven normal cervical tissue samples collected from a random population (used as control) and in cervical tissue samples from patients with Carcinoma Cervix. Expression of physiological microRNA RNUB6 (used as housekeeping microRNA) was examined both in carcinoma and normal cervical tissue.

Every patient was evaluated clinically and with MRI (Magnetic Resonance Imaging) pelvis before starting the treatment. All the patients received standard therapy of External Beam Radiotherapy (EBRT) by intensity‐modulated radiotherapy technique to a dose of 45‐50.4 Gy in 25‐28 fractions with concurrent chemotherapy of weekly Injection Cisplatin (40 mg/m^2^) or carboplatin (Area under curve = 2) followed by high dose rate brachytherapy. The entire treatment was completed within 8 weeks in all the patients. The patients were re‐evaluated for the clinical response after 3 months of completion of treatment with pelvic examination and MRI pelvis scan using RECIST (Response evaluation criteria in solid tumors) 1.1 criteria.^18^ Patients who had no residual disease were classified as clinical responders (CR) and patients who had residual or progressive disease were classified as non‐responders (NR).

Expression of six microRNAs (microRNA‐9 5p, ‐31 3p, ‐100 5p, ‐125a 5p, ‐125b 5p, and ‐200a 5p) were studied in complete responders (CR) and nonresponders (NR).

Mann Whiney *U* test was used to study the significant difference between the expression of microRNA between complete clinical responder and nonresponder (NR) to standard therapy. Statistical analysis was done by IBM (International Business Machines Corporation) Statistical Package for the Social Sciences (SPSS) version 26.

In accordance with the journal's guidelines, we will provide our data for the reproducibility of this study in other centers if such is requested.

## 3 RESULTS

During the study period six out of 38 patients were lost to follow up. So, we present here the analysis of results for 32 patients only. The clinic pathological characteristics of the patients in the study are described in Table 1. The median age of the patients was 53 years (range 35‐71 years). On 3 months follow up, with clinical examination and MRI pelvis imaging, 24 patients (75%) showed complete response (CR) while 8 patients (25%) had nonresponse to standard therapy (NR).

**TABLE 1:** Patient characteristics

Patient characteristics (*n* = 32)	*N*, (%)
Age [years]	
[Median, range]	53 [35‐71]
FIGO stage	
IB	0 [0%]
IIA	3 [9%]
IIB	16 [50%]
IIIA	2 [6%]
IIIB	11 [35%]
IVA	0 [0%]
Histology	
Squamous cell carcinoma:	30 [94%]
Adeno carcinoma:	2 [6%]
EBRT Dose [Gy]	
Median	50.4 Gy
Range	45–50.4
Concurrent chemotherapy	
Cisplatin	30 [94%]
Carboplatin	2 [6%]
No of cycle of chemotherapy	
5 cycle	19 [59%]
6 cycle	13 [41%]
Response to treatment	
Complete response (CR)	24 [75%]
Non response (NR)	8 [25%]

**FIGURE 1:**
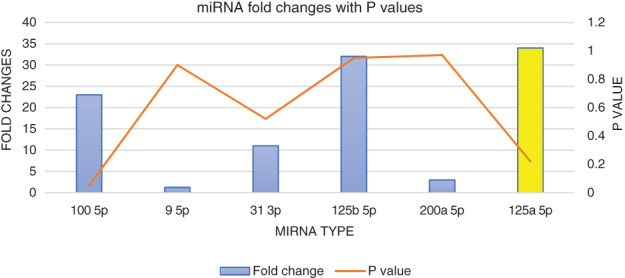
Graphical representation miRNA fold changes with respective *p* value in patients with complete responders compared with patients with non‐response to standard therapy in carcinoma cervix. microRNA in blue color shows upregulation and microRNA in yellow color shows downregulation

When microRNA expression was compared in complete responder (CR) to nonresponder (NR) (Table 2, Figure 1), it was seen that expression of microRNA‐100 5p was upregulated 23‐fold in complete responders (CR) compared to nonresponders (NR), which showed a trend towards statistical significance (*p* value 0.05). The following microRNA were upregulated in complete responders compared to nonresponders but was not statistically significant: microRNA‐9 5p (1.2‐fold, *p* value 0.90), microRNA‐31 3p (11‐fold, *p* value 0.52), microRNA‐125b (32‐fold, *p* value 0.95), and microRNA‐200a 5p (3‐fold, *p* value 0.97). However, microRNA‐125a 5p was downregulated 34‐fold in complete responders compared to nonresponders (*p* value 0.22) which was also statistically nonsignificant.

**TABLE 2:** Expression of microRNA in complete responders (CR) compared to non‐responders (NR) to chemoradiation in locally advanced carcinoma cervix

microRNA	Expression	Fold change	*p* Value
100 5p	Upregulated	23‐fold	0.05
9 5p	Upregulated	1.2‐fold	0.90
31 3p	Upregulated	11‐fold	0.52
125b 5p	Upregulated	32‐fold	0.95
200a 5p	Upregulated	3‐fold	0.97
125a 5p	Downregulated	34‐fold	0.22

“The association between expression of the six miRNAs in complete responders and non‐responders were tested by Pearson Correlation test. There was no correlation found between the expression of these six miRNAs in complete responders and non‐responders.”

## 4 DISCUSSION

Several studies have reported microRNA as potential biomarkers in different types of cancer such as lung cancer, colorectal cancer, breast cancer, and chronic lymphocytic leukemia.^19^, ^20^, ^21^ However, there are very few studies reporting microRNA as predicting biomarkers in carcinoma cervix.

microRNA‐100 expression was studied by Li et al, which showed microRNA‐100 expression gradually reduced from low‐grade to high‐grade carcinoma in situ to cervical cancer tissues, and a significant decrease in HPV positive cervical cancer cells and its effect is mediated through PLK1 protein.^22^ PLK1 inhibition causes cells to accumulate in the G2/M phase of the cell cycle and results in increased radiosensitivity of cells.^23^ Yang et al showed upregulation of microRNA‐100 increases radiosensitivity in colorectal cancer.^24^ Chondrosarcoma cells exhibit decreased expression of microRNA‐100 resulting in cisplatin resistance through mTOR pathway.^25^ microRNA‐100 also increases sensitivity to docetaxel chemotherapy in lung adenocarcinoma cells through PLK1 protein.^26^ Also, the expression of microRNA‐100 affects small cell lung cancer cell survival and chemoresistance by downregulating HOXA1 gene.^27^ Overall, all studies suggested that the expression of microRNA‐100 increases the sensitivity of cancer cells towards chemoradiotherapy. Hence, there is a high probability that microRNA‐100 is upregulated in patients who respond to chemo‐radiotherapy than patients who do not respond to the treatment of chemo‐radiotherapy. In our study, we showed that expression of microRNA‐100 5p is upregulated 23‐fold in complete responders (CR) to non‐responders (NR), which showed a trend towards statistical significance (*p* value 0.05).

Hu et al reported microRNA‐200a and microRNA‐9 to have predictive value for the survival of cervical cancer patients.^16^ They suggested microRNA‐200a may affect the metastatic potential of cervical carcinoma cells by suppressing multiple genes that regulate the motility of cells. Yu et al have reported microRNA‐200a to play a role in chemoresistance by downregulating TP53INP1 and YAP1 in human breast cancer.^28^ Similarly, microRNA‐9 enhances the sensitivity of cells to ionizing radiation by suppression of NFκB1.^29^ So, there is a probability that microRNA‐200a and microRNA‐9 will be upregulated in patients who respond to chemo‐radiotherapy. In our study, we found microRNA‐200 5p was upregulated 3‐fold and microRNA‐9 5p was upregulated 1.2‐fold in complete responder (CR) than nonresponders (NR).

microRNA‐125a inhibited the proliferation of Non‐Small Cell Lung cancer cells and promoted their apoptosis, thus reduces the chemoresistance of Non‐small Cell Lung Cancer.^30^ This suggests that microRNA‐125a might be upregulated in patients responding to chemotherapy. But, in our study microRNA‐125a is downregulated in complete responders (CR) which was not statistically significant (*p* value 0.22). This result of the downregulation of microRNA‐125a could not be explained by evidence of any reported studies, which may be specific to our study population.

Shiiba et al reported that microRNA‐125b plays a role in decreased proliferation rate, enhanced radiosensitivity of squamous cell cancer cells of the oral cavity through decreased ICAM2 messenger RNA expression.^31^ Our study showed that microRNA‐125b 5p is upregulated 32‐ fold (though not statistically significant, *p* value 0.95) in complete responders (CR).

microRNA‐31expression is associated with apoptosis and increased sensitivity of triple‐negative breast cancer cells to ionizing radiation and chemotherapeutics by down‐regulation of PKCɛ resulting in impaired NF‐κB signaling.^32^ Our study showed that the expression of microRNA‐31 3p is upregulated 11‐fold (though statistically not significant, *p* value 0.52) in complete responders (CR) to chemoradiotherapy in locally advanced cervical cancer patients.

Though Hu et al and Pedroza‐Torres et al showed microRNA‐9, ‐31, ‐100, ‐125a, ‐125b, and ‐200a expression was associated with clinical outcome significantly, our study has shown that only microRNA‐100 5p was upregulated in patients with locally advanced cervical cancer who responds to chemoradiotherapy, which showed a trend towards statistical significance (*p* value 0.05).^15^, ^16^ microRNA‐9 5p, ‐31 3p, ‐125b, and ‐200a 5p was upregulated and microRNA‐125a 5p was downregulated in complete responders, though the result was not statistically significant. This result of statistical insignificance may be due to the small sample size of our study population.

We propose that the results of our study should be validated in a study population with a larger sample size so that they could be used as predictors of response to chemoradiotherapy in locally advanced carcinoma cervix.

## CONFLICT OF INTERESTS

The authors have stated explicitly that there are no conflicts of interest in connection with this article.

## AUTHOR CONTRIBUTIONS

All authors contributed significantly to manuscript and inagreement with content of manuscript. **Soumitra Barik:** Conceptualization; data curation; formal analysis; investigation; methodology; project administration; supervision; visualization; writing‐original draft; writing‐review and editing. **Swarupa Mitra:** Conceptualization; data curation; formal analysis; investigation; methodology; project administration; supervision; validation; visualization; writing‐original draft; writing‐review and editing. **Moushumi Suryavanshi:** Conceptualization; data curation; formal analysis; investigation; methodology; project administration; resources; supervision; validation; visualization; writing‐original draft; writing‐review and editing. **Abhinav Dewan:** Conceptualization; data curation; formal analysis; funding acquisition; investigation; methodology; project administration; supervision; validation; visualization; writing‐original draft; writing‐review and editing. **INDERJEET WAHI:** Conceptualization; data curation; formal analysis; investigation; methodology; project administration; supervision; validation; visualization; writing‐original draft; writing‐review and editing. **DUSHYANT KUMAR:** Conceptualization; data curation; formal analysis; funding acquisition; investigation; methodology; project administration; supervision; validation; visualization; writing‐original draft; writing‐review and editing. **Manindra Mishra:** Conceptualization; data curation; formal analysis; investigation; methodology; project administration; supervision; validation; visualization; writing‐original draft; writing‐review and editing. **Gayatri Vishwakarma:** Conceptualization; data curation; formal analysis; investigation; methodology; project administration; supervision; validation; visualization; writing‐original draft; writing‐review and editing.

## ETHICAL STATEMENT

The study design was approved by the institutional ethics review board before starting of study. Informed written consent was taken from every patient before recruitingAQ5 in the study. We have not reproduced any material from any other source.

This study and article submitted to *Cancer Reports* has been done in accordance to the guidelines of the journal and that is has been performed in an ethical and responsible way, with no research misconduct, which includes, but is not limited to data fabrication and falsification, plagiarism, image manipulation, unethical research, biased reporting, authorship abuse, redundant or duplicate publication, and undeclared conflicts of interest.

## CONFLICT OF INTEREST

Authors declare no conflict of interests.

## Data Availability

The data that support the findings of this study are available from the corresponding author upon reasonable request.

## References

[1] Bray F, Ferlay J, Soerjomataram I, Siegel RL, Torre LA, Jemal A. Global cancer statistics 2018: GLOBOCAN estimates of incidence and mortality worldwide for 36 cancers in 185 countries. CA Cancer J Clin. 2018;68(6):394‐424. 10.3322/caac.21492. 30207593

[2] Rojas‐Espaillat LA, Rose PG. Management of locally advanced cervical cancer. Curr Opin Oncol. 2005;17(5):485‐492. 10.1097/01.cco.0000174049.14515.8d. 16093801

[3] Bartel DP. MicroRNAs: genomics, biogenesis, mechanism, and function. Cell. 2004;116(2):281‐297. 10.1016/s0092-8674(04)00045-5. 14744438

[4] He L, Hannon GJ. MicroRNAs: small RNAs with a big role in gene regulation. Nat Rev Genet. 2004;5(7):522‐531. 10.1038/nrg1379. 15211354

[5] Mendell JT. MicroRNAs: critical regulators of development, cellular physiology and malignancy. Cell Cycle. 2005;4(9):1179‐1184. 10.4161/cc.4.9.2032. 16096373

[6] Lim LP, Lau NC, Garrett‐Engele P, et al. Microarray analysis shows that some microRNAs downregulate large numbers of target mRNAs. Nature. 2005;433(7027):769‐773. 10.1038/nature03315. 15685193

[7] Lewis BP, Burge CB, Bartel DP. Conserved seed pairing, often flanked by adenosines, indicates that thousands of human genes are microRNA targets. Cell. 2005;120(1):15‐20. 10.1016/j.cell.2004.12.035. 15652477

[8] Miska EA. How microRNAs control cell division, differentiation and death. Curr Opin Genet Dev. 2005;15(5):563‐568. 10.1016/j.gde.2005.08.005. 16099643

[9] Calin GA, Croce CM. MicroRNA signatures in human cancers. Nat Rev Cancer. 2006;6(11):857‐866. 10.1038/nrc1997. 17060945

[10] Kent OA, Mendell JT. A small piece in the cancer puzzle: microRNAs as tumor suppressors and oncogenes. Oncogene. 2006;25(46):6188‐6196. 10.1038/sj.onc.1209913. 17028598

[11] Johnson CD, Esquela‐Kerscher A, Stefani G, et al. The let‐7 microRNA represses cell proliferation pathways in human cells. Cancer Res. 2007;67(16):7713‐7722. 10.1158/0008-5472.CAN-07-1083. 17699775

[12] Wang X, Tang S, Le S, et al. Aberrant expression of oncogenic and tumor‐suppressive microRNAs in cervical cancer is required for cancer cell growth. PLoS One. 2008;3(7):e2557. 10.1371/journal.pone.0002557. 18596939 PMC2438475

[13] Li Y, Wang F, Xu J, et al. Progressive miRNA expression profiles in cervical carcinogenesis and identification of HPV‐related target genes for miR‐29. J Pathol. 2011;224(4):484‐495. 10.1002/path.2873. 21503900

[14] Wang X, Wang H, Li Y, et al. microRNAs are biomarkers of oncogenic human papillomavirus infection. Proc Natl Acad Sci U S A. 2014;111(11):4262‐4267. 10.1073/pnas.1401430111. 24591631 PMC3964092

[15] Hu X, Schwarz JK, Lewis JS Jr, et al. A microRNA expression signature for cervical cancer prognosis. Cancer Res. 2010;70(4):1441‐1448. 10.1158/0008-5472.CAN-09-3289 [published Online First: 2 Feb 2010]. 20124485 PMC2844247

[16] Pedroza‐Torres A, Fernández‐Retana J, Peralta‐Zaragoza O, et al. A microRNA expression signature for clinical response in locally advanced cervical cancer. Gynecol Oncol. 2016;142(3):557‐565. 10.1016/j.ygyno.2016.07.093. 27423381

[17] Livak KJ, Schmittgen TD. Analysis of relative gene expression data using real‐time quantitative PCR and the 2(‐Delta Delta C[T]) method. Methods. 2001;25(4):402‐408. 10.1006/meth.2001.1262. 11846609

[18] Eisenhauer EA, Therasse P, Bogaerts J, et al. New response evaluation criteria in solid tumours: revised RECIST guideline (version 1.1). Eur J Cancer. 2009;45(2):228‐247. 10.1016/j.ejca.2008.10.026. 19097774

[19] Chen X, Ba Y, Ma M, et al. Characterization of microRNAs in serum: a novel class of biomarkers for diagnosis of cancer and other diseases. Cell Res. 2008;18(10):997‐1006. 10.1038/cr.2008.282. 18766170

[20] Zhao H, Shen J, Medico L, Wang D, Ambrosone CB, Liu S. A pilot study of circulating miRNAs as potential biomarkers of early stage breast cancer. PLoS One. 2010;5(10):e13735. 10.1371/journal.pone.0013735. 21060830 PMC2966402

[21] Calin GA, Ferracin M, Cimmino A, et al. A MicroRNA signature associated with prognosis and progression in chronic lymphocytic leukemia. N Engl J Med. 2005;353(17):1793‐1801. 10.1056/NEJMoa050995. 16251535

[22] Li BH, Zhou JS, Ye F, et al. Reduced miR‐100 expression in cervical cancer and precursors and its carcinogenic effect through targeting PLK1 protein. Eur J Cancer. 2011;47(14):2166‐2174. 10.1016/j.ejca.2011.04.037. 21636267

[23] Lund‐Andersen C, Patzke S, Nähse‐Kumpf V, Syljuasen RG. PLK1‐inhibition can cause radiosensitization or radioresistance dependent on the treatment schedule. Radiother Oncol. 2014;110(2):355‐361. 10.1016/j.radonc.2013.12.014. 24502970

[24] Yang XD, Xu X, Zhang S, et al. Role of miR‐100 in the radioresistance of colorectal cancer cells. Am J Cancer Res. 2015;5(2):545‐559. 25973296 PMC4396051

[25] Zhu Z, Wang C, Zhang Y, Nie L. MicroRNA‐100 resensitizes resistant chondrosarcoma cells to cisplatin through direct targeting of mTOR. Asian Pac J Cancer Prev. 2014;15(2):917‐923. 10.7314/apjcp.2014.15.2.917. 24568519

[26] Feng B, Wang R, Chen L. MiR‐100 resensitizes docetaxel‐resistant human lung adenocarcinoma cells (SPC‐A1) to docetaxel by targeting Plk1. Cancer Lett. 2012;317(2):184‐191. 10.1016/j.canlet.2011.11.024. 22120675

[27] Xiao F, Bai Y, Chen Z, et al. Downregulation of HOXA1 gene affects small cell lung cancer cell survival and chemoresistance under the regulation of miR‐100. Eur J Cancer. 2014;50(8):1541‐1554. 10.1016/j.ejca.2014.01.024. 24559685

[28] Yu S, Yang L, Hong Q, Kuang X, Di G, Shao Z. MicroRNA‐200a confers chemoresistance by antagonizing TP53INP1 and YAP1 in human breast cancer. BMC Cancer. 2018;18(1):74. 10.1186/s12885-017-3930-0. 29329575 PMC5766993

[29] Arora H, Qureshi R, Jin S, Park A, Park W. miR‐9 and let‐7g enhance the sensitivity to ionizing radiation by suppression of NFκB1. Exp Mol Med. 2011;43(5):298‐304. 10.3858/emm.2011.43.5.031. 21464588 PMC3104252

[30] Li C, Zhai W, Wan L, et al. MicroRNA‐125a attenuates the chemoresistance against ubenimex in non‐small cell lung carcinoma via targeting the Aminopeptidase N signaling pathway. J Cell Biochem. 2020;121(2):1716‐1727. 10.1002/jcb.29407. 31595566

[31] Shiiba M, Shinozuka K, Saito K, et al. MicroRNA‐125b regulates proliferation and radioresistance of oral squamous cell carcinoma. Br J Cancer. 2013;108(9):1817‐1821. 10.1038/bjc.2013.175. 23591197 PMC3658524

[32] Körner C, Keklikoglou L, Bender C, Wörner A, Münstermann E, Wiemann S. MicroRNA‐31 sensitizes human breast cells to apoptosis by direct targeting of protein kinase C ɛ (PKCɛ). J Biol Chem. 2013;288(12):8750‐8761. 10.1074/jbc.M112.414128. 23364795 PMC3605692

